# Practical Socialism
*"Practical Socialism: Essays on Social Reform." By the Rev. and Mrs. Samuel A. Barnett. Longmans, Green, and Co.


**Published:** 1889-04-13

**Authors:** 


### Practical Socialism.*

These essays, thirteen in number, were written at different intervals during the fifteen years of residence of the authors in East London. In the first article, headed "The Poverty of the Poor," the question is asked, "Why cannot and does not each man, woman, and child attain to the normal standard of robustness?" We are glad to see that it is not attributed to drink alone, and we fully agree with the writer, Mrs. S. A. Barnett, that "the working classes, as a rule, do not drink." The answer given to the question is one word, "Poverty," which means scarcity of food. It is calculated that each man requires 20oz. of solid food per day —*i.e.*, 16oz. of carbonaceous, or strength-giving food, and 4oz. of nitrogenous or flesh-forming food. The woman should eat 12oz. of carbonaceous and 3oz. of nitrogenous food, and when doing rough work another ounce of the latter per day. On the average, children require 8oz. of carbonaceous and 2oz. of nitrogenous food per day. For a man and woman with eight children, 92oz. of carbonaceous and 23oz. of nitrogenous material are required. Tables are given of different dietaries which approximate to this standard, at a cost of 2s. 5d. or 2s. 4d. per diem, or a total of 16s. 4d. at the week's end. This leaves the labourer earning £1 per week 3s. 8d. with which to provide rent, clothing, firing, and all other necessaries. This cannot be done, so food is stinted, and recourse is had to alcohol by the father to "pick him up and hold him together." But the dietaries shown by the authoress are too little for the average labouring man. Twenty-six ounces, one-third being animal matter, is the least the average labouring man should consume. Mrs. Barnett's diets are shown chemically, but dietaries based on chemical constituents are more or less fallacious. Vital processes are incapable of chemical explanation. The saying that one man's meat is another man's poison is correct. There are persons who cannot eat beef, and there are others who cannot even touch mutton. Valentin, from experiments on himself, found that his daily consumption was 6lb. of solid and liquid food; but Cornaro lived on 12oz. of solid food, with 14oz. of light wine. We do not quite understand what Mrs. Barnett means by carbonaceous or strength-giving food. Food is certainly chiefly applied in two ways. Carbonaceous food is principally consumed in the production of animal heat, nitrogenous is chiefly appropriated for the use of animal tissues. But both are required to give strength. Again, the more warmly we are clad, the less urgent the appetite for carbonaceous food, for the loss of heat by cooling is diminished, and therefore the amount to be derived from food is lessened.

All this respecting poverty and its attendant want being the cause of the mental and physical degeneration of the poor is bad enough, but it is only half the truth. It is not want of sufficient food alone which causes degradation. There are the unsanitary conditions under which so many exist, recently so vividly portrayed by the *Sunday Times*, and to which, in our opinion, sufficient importance has not been attached by the authoress of the essay under notice. Then there is the prevalent inherited scrofulous and specific taints. Such taints might certainly not always develop were the poor well fed, and living in good sanitary localities, with necessary attention to personal hygiene. But all this seems a sealed book to our authoress. Then, again, much evil must be attributed to too early marriages among the poor. In this respect they do not practise the abstinence and caution to which the middle classes are compelled. Our authoress brings forward as a type a family of eight children. We fancy if an employé—a clerk, say—on two or three hundred per annum were blessed with eight olive branches, he could not with greater truth than the labourer or artisan, apply the lines

> "Happy the man who, void of care and strife,
> In silken or in leather purse retains
> The splendid shilling."

The relief funds of the poor is treated of by the Rev. S. A. Barnett, who, after remarking on the failure of the Mansion House Relief Funds, quotes a Lord Mayor (whose name does not appear) to the effect that "spasmodic assistance given by the public in answer to special appeals is really useless." In Whitechapel a labour test was applied, The labourers were offered street-sweeping, and those who were used only to indoor work were put to whitewashing, window-cleaning, or tailoring. The women were given needlework. "When it was known that work was to be offered there arose among the ne'er-do-wells great indignation. 'Call this charity!' they exclaimed. Many finding they could not get relief without doing work did not persist in their application." Many notorious evil-livers got, by a good story, the relief denied to others. "In the face of all this experience it is not extravagant to say that the means of relief used last winter (1886) developed the causes of poverty." But poverty in London increases. As for the cure, the author has little better to offer than the following: It "must be worked by slow means, which will take account of the whole nature of man, which will consider the future to be as important as the present, and which will win by waiting." Then we are told that it is the practice of living in pleasant places which impoverishes the poor, and that the precept "every one should live over his shop" has a very direct bearing on the subject. Next, that the habit of getting the most for one's money provokes that competition by which so many are trampled under foot. In an essay on "Passionless Reformers," Henrietta O. Barnett asks the question, "How can these people be raised to enjoy spiritual life?" and, in reply, suggests gifts of flowers, high-class music (both instrumental and vocal) given in schoolrooms, mission-rooms, and if possible in churches, quiet afternoons in the country, but not treats where numbers bring wild excitement, the stories of great lives and of other religions simply told, churches open in the daytime to the poor, and public picture galleries. We fear it would be long before the uneducated and mentally and physically deteriorated poor could be brought to seek solace in such manners, even if the means were ready at hand. Such ideas seem utterly Utopian at the present time.

A better essay is by the Rev. Mr. Barnett, on "Town Councils and Social Reform." He states some way must be found which, without pauperising, without affecting the spirit of energy and independence, shall give to the inhabitants of our great towns the surroundings which will increase joy and develop life. The first need is better dwellings. Insanitary conditions and high rents are the points to which consideration must be directed. The first practical work is to raise Town Councils to a sense of their powers, to make them feel that the reason of their being is not political, but social; that their duty is not to protect the pockets of the rich, but to save the people. All this is very well, but we fancy Town Councils generally are willing and ready to assist the poor, but, like Shakespeare's apothecary, their own comparative poverty, and not their will, forbids the desired course. Perhaps County Councils will prevail.

An essay on Young Women in our Workhouses, which first appeared in *Macmillan's Magazine*, by Henrietta O. Barnett, deals with young single women who are found in the workhouses for one of three reasons: first, for shelter when about to become mothers; secondly, for the evil results of profli-

* "Practical Socialism: Essays on Social Reform." By the Rev. and Mrs. Samuel A. Barnett. Longmans, Green, and Co.

gacy; thirdly, they choose to enter rather than sin or starve. It is a common idea that the only way of helping young women is to send them to homes, and homes are very valuable as giving girls the opportunity of regaining a character. But Mrs. Barnett thinks that around every workhouse a committee of ladies might be formed, with the view of visiting the inmates and obtaining employment for the deserving.

Now, we do not think that the essays which form the volume entitled "Practical Socialism" strike at the root of the evil of poverty which exists—a poverty which is more destructive than the evanescent results of war or pestilence. Twenty years ago the brothers Mayhew wrote on "London Labour and the London Poor," and their writings are applicable to the present hour. And this notwithstanding that we count no less than 54 different appeals (not including hospitals) on behalf of the poor in this (Christmas) week's newspapers. The cause of low wages, or in other words of poverty, was long ago explained by Malthus and Mill as over-population. We have referred before to early unions among the poor. We do not suppose that in this country the Legislature would ever interfere with the view of limitation; but this is a matter which should be pressed on the attention of the people by clergymen, social reformers, and philanthropists. Next, there is an improved and extended system of emigration which should be established. Some legislative measures might also be considered which would tend to check the annually increasing influx of foreigners into Great Britain, and especially into London. Former generations knew little about the laws of health. They built their streets narrow, their chambers small; they huddled their buildings as closely together as possible, leaving few open spaces. Provision of suitable homes for the poor is a legitimate use to which public or local funds may be applied. Without good sanitary surroundings, there can be no sound health, and bad health is synonymous with poverty. Moreover, the poor have a right to demand labour, and fair wages therefore. No man in this London, or in the country, ought to be allowed to be idle if willing to work. Government should provide work for those not in the employment of private firms. And this, with the multitudinous requirements of the land, would not appear to be difficult. For the sick and infirm there are workhouses, infirmaries, and hospitals, also outdoor relief, all of which, if not sufficient, should be increased. As for the large class of loafers, those who prefer alms to honest work, the sooner they understand nothing will be done for them the better. The sooner they understand the fallacy of their idea, as recorded by the Rev. Mr. Barnett, "The fund is a charity, and we ought not to work for it," the sooner will poverty in London decrease. "The poor ye have always with you," was said 2,000 years ago. There are poor in every land, and under every form of government. The position must be accepted. Amelioration can only be attained—not cure. The general practical means are as noted above. All that can be done by private societies, committees, or individuals is a mere drop in the ocean of requirements. In the meantime, as the *avant-courent* of better things, it is satisfactory to note the reply of the Mayor of Newcastle, when recently interviewed by the correspondent of the *Sunday Times*. In answer to a question as to the condition of the people, the mayor said: "There is an abundance of work, and very little real poverty. All our labouring classes are well employed in various trades, and one may say we have 'no complaining in our streets.' Everyone may work that will—and the unwilling here are very exceptional. The poorer classes are better educated, and, with us, that means that they are more thrifty."

